# JNK Pathway Activation Is Controlled by Tao/TAOK3 to Modulate Ethanol Sensitivity

**DOI:** 10.1371/journal.pone.0050594

**Published:** 2012-12-05

**Authors:** David Kapfhamer, Ian King, Mimi E. Zou, Jana P. Lim, Ulrike Heberlein, Fred W. Wolf

**Affiliations:** 1 The Ernest Gallo Clinic and Research Center, University of California San Francisco, Emeryville, California, United States of America; 2 Department of Anatomy, Program in Neuroscience, University of California San Francisco, San Francisco, California, United States of America; Thomas Jefferson University, United States of America

## Abstract

Neuronal signal transduction by the JNK MAP kinase pathway is altered by a broad array of stimuli including exposure to the widely abused drug ethanol, but the behavioral relevance and the regulation of JNK signaling is unclear. Here we demonstrate that JNK signaling functions downstream of the Sterile20 kinase family gene *tao*/*Taok3* to regulate the behavioral effects of acute ethanol exposure in both the fruit fly *Drosophila* and mice. In flies *tao* is required in neurons to promote sensitivity to the locomotor stimulant effects of acute ethanol exposure and to establish specific brain structures. Reduced expression of key JNK pathway genes substantially rescued the structural and behavioral phenotypes of *tao* mutants. Decreasing and increasing JNK pathway activity resulted in increased and decreased sensitivity to the locomotor stimulant properties of acute ethanol exposure, respectively. Further, JNK expression in a limited pattern of neurons that included brain regions implicated in ethanol responses was sufficient to restore normal behavior. Mice heterozygous for a disrupted allele of the homologous *Taok3* gene (*Taok3Gt*) were resistant to the acute sedative effects of ethanol. JNK activity was constitutively increased in brains of *Taok3Gt/+* mice, and acute induction of phospho-JNK in brain tissue by ethanol was occluded in *Taok3Gt/+* mice. Finally, acute administration of a JNK inhibitor conferred resistance to the sedative effects of ethanol in wild-type but not *Taok3Gt/+* mice. Taken together, these data support a role of a TAO/TAOK3-JNK neuronal signaling pathway in regulating sensitivity to acute ethanol exposure in flies and in mice.

## Introduction

Alcohol is a highly popular psychoactive drug that humans have manufactured and consumed since prehistoric times. However, alcohol abuse is widespread and it imposes high social and monetary costs on society [1]. The efficacy of behavioral and medical interventions for alcohol abusers may be improved by understanding the basic biological mechanisms by which alcohol coopts normal body functions to lead to maladaptive behaviors. Previous studies demonstrated that a low level of response to the inebriating effects of alcohol is associated with an increased risk for developing alcohol use disorders [2], [3]. This implies that genes that govern responding to acute alcohol exposure may contribute to alcohol abuse. Understanding the genetic basis of the pharmacological actions of alcohol may thus provide new approaches to treatment of alcohol use disorders. The development of sophisticated assays that model aspects of alcoholism in genetically tractable organisms such as the mouse and the fruit fly *Drosophila* has greatly accelerated the identification of genes that impact alcohol responses, and some of these studies have led to preclinical and clinical trials for new treatments [4], [5], [6].

Mitogen activated protein kinases (MAPKs) comprise multi-tiered signal transduction cascades involved in sensing diverse extracellular stimuli including growth factors, cytokines and environmental stressors. MAPK activation induces changes in transcription, cellular morphology, differentiation, proliferation, and in cell death [7], [8]. Three partially overlapping MAPK pathways exist that are defined by the p38, extracellular signal regulated kinase (ERK), and c-jun N-terminal kinase (JNK) families [9], [10], [11].

Ethanol modulates MAPK signaling in multiple tissues, including the brain [9], [12], [13], [14]. In the fruit fly *Drosophila* and in mice, inhibition of the EGFR/ERK pathway increases sensitivity to ethanol-induced sedation [4], [15], and ERK also regulates ethanol sensitivity of the camouflage response in zebrafish [16], [17]. Chronic ethanol consumption and ethanol withdrawal induces ERK-dependent changes in synaptic plasticity in the rat dorsolateral striatum [18], and pharmacological inhibition of ERK signaling in mice can alter operant responding for ethanol [19]. Additionally, both ERK and JNK are activated in the basolateral amygdala in response to ethanol-associated cues [20]. Long-term exposure to ethanol can also induce JNK and p38 activity in the brain [21]. It is not known if the activity of either the JNK or the p38 pathway in the brain is functionally relevant for the behavioral effects of ethanol.

*Drosophila tao* promotes sensitivity to the locomotor stimulating effects of acute ethanol exposure [22]. *tao* encodes a serine-threonine kinase of the GCK-VIII subfamily of the Ste20p family, characterized by a highly conserved N-terminal kinase domain and a C-terminal region that regulates cytoskeletal dynamics [23], [24], [25], [26], [27], [28]. Fly TAO is homologous to mammalian TAOK1, TAOK2 and TAOK3 [23], [29], [30]. Cell culture experiments support a role for mammalian TAOK in MAPK signaling through the p38 and/or JNK pathways. All three TAOK proteins can activate p38 [31], [32], [33]. Further, JNK signaling can be activated by TAOK1 and TAOK2, whereas it can be inhibited by TAOK3 [23], [27], [30], [31].

To identify TAOK signaling pathways we searched for genetic interactions between fly TAO and key constituents of candidate MAPK pathways. Here we demonstrate that JNK is negatively regulated by TAO/TAOK3 *in vivo*, and we provide evidence for a neuronal role for JNK signaling in the behavioral effects of acute ethanol exposure. Support for these findings come from two evolutionarily divergent model organisms, the mouse and the fruit fly, suggesting that the relationship between TAO/TAOK3 and JNK in ethanol behavioral responses is conserved.

## Materials and Methods

### Ethics Statement

All animal protocols were approved by the Ernest Gallo Clinic and Research Center (EGCRC) Institutional Animal Care and Use Committee (approval number 09.11.197).

### *Drosophila* Strains and Behavior

Flies were maintained on standard cornmeal/molasses/yeast media at 25°C and 70% relative humidity with an approximately 16 hr/8 hr light/dark schedule. Mutant strains were obtained from the NIG Kyoto Stock Center (*puc^NP1311^*), the Harvard Exelixis collection (*bsk^EY01915^*), and the Bloomington *Drosophila* Stock Center. All strains were outcrossed for at least five generations to the *w^1118^* Berlin genetic background. Behavior experiments used groups of 20 genetically identical male flies, and experiments were repeated on multiple days. Ethanol-induced locomotion assays were performed as described previously [34], [35]. Flies were exposed to 33% ethanol vapor (50∶100 arbitrary flow units of ethanol:humidified air at a total flow of 5.5 L/min). Movies of ethanol-exposed flies were captured using QuickTime, and analyzed using a modified version of DIAS motion tracking software. The average speed of a population of 20 flies was determined at 14 intervals of 15 sec duration over the course of a 21 min exposure, and plotted versus time of exposure. Ethanol-induced locomotor activity was quantified as the area under the curve during the hyperactive phase. Ethanol absorption was measured in populations of 20 male flies, exposed to 33% ethanol vapor for 21 minutes. Flies were flash frozen, homogenized, and assayed for ethanol content using an alcohol dehydrogenase-based spectrophotometric assay (Diagnostic Chemicals, Ltd).

### *Drosophila* Immunohistochemistry

Brains were dissected in PBS, fixed for 30 min at room temperature in PBS with 4% formaldehyde, washed twice in PBS with 0.3% triton X-100 (PBT), and blocked in PBT with 5% normal goat serum. Samples were incubated with primary antibody overnight, diluted as follows in PBT with 5% normal goat serum: anti-Fasciclin II 1∶200 (1D4), anti-repo 1∶10 (8D12, Developmental Studies Hybridoma Bank, Iowa), anti-GFP 1∶1000 (Invitrogen), anti-TH 1∶100 (Immunostar). After treatment with primary antibody, samples were washed three times in PBT and incubated overnight with fluorescently labeled secondary antibody diluted 1∶500 (Molecular Probes). Samples were washed three times in PBT before mounting in Vectashield (Vector Labs).

### Generation of *Taok3* genetrap (*Taok3Gt*) Mice

C57BL/6J-derived blastocysts were injected with the RRK451 genetrap line ES cells, obtained from Bay Genomics (www.genetrap.org), according to standard protocols [36]. The genetrap line was generated on a 129/OlaHsd (agouti) background. Several chimeras were crossed to wild-type C57BL/6J mice and germline transmission of the targeted allele in progeny was confirmed by identifying agouti pups. Backcrosses of the resulting F1 mice (*Taok3Gt^129^/+^B6^* X C57BL/6J) for 5 or more generations were done to generate *Taok3Gt/+* heterozygous and wild-type controls for all studies, with two exceptions: studies to address the effects of SP600125 on ethanol consumption and clearance were performed on C57BL/6J mice obtained from Jackson Laboratory (Bar Harbor, ME), and locomotor stimulation experiments were performed on a C57BL/6J X 129/SvJ F1 background. Ethanol has been reported to weakly stimulate activity in C57BL/6J mice [37]. For this reason, *Taok3Gt^B6^/+^B6^* were crossed to 129/SvJ mice and the resulting F1 progeny were used for ethanol-induced locomotor stimulation experiments.

### Mouse Genotypic Analysis

Mice were genotyped by PCR with DNA isolated from tail biopsies using standard protocols. The following primers were used to amplify a fragment of the genetrap insertion: (forward: 5′ TTATCGATGAGCGTGGTGGTTATGC-3′; reverse: 5′-GCGCGTACATCGGGCAAATAATATC-3′).

### Quantitative RT-PCR (qPCR)

Total RNA was isolated from whole brain tissue using Trizol® reagent (Invitrogen, Carlsbad, CA, USA) according to the manufacturer’s instructions and was treated with RNase-free DNase (Promega, Madison, WI, USA) to remove genomic DNA contamination. cDNA was synthesized from 1 µg of total RNA using reverse transcription reagents from Applied Biosystems (Foster City, CA, USA). Following synthesis, cDNA was diluted 1∶10 in water. TaqMan qPCR was performed using standard thermal cycling conditions on an ABI PRISM 7900 Sequence Detection System (Applied Biosystems). Amplification reactions contained 5 µl of cDNA template, 1× Universal PCR Master Mix, 100 nM each of forward and reverse primers, and 200 nM of FAM-labeled probe in a final volume of 10 µl. Taqman probesets used in this study were *Taok3* (spanning exons 10–11): Mm001195800_m1, rodent *Gapdh*: 4352932E, *Drosophila puc*: Dm02135504_m1, *Drosophila RpL32*: Dm02151827_g1 (Applied Biosystems). Data were analyzed using the comparative C_T_ method (Applied Biosystems user bulletin #2).

### Protein Analysis

Protein extracts were derived from whole brain tissue in RIPA buffer containing phosphatase inhibitor (Sigma Aldrich, Inc., St. Louis, MO, USA) using standard techniques. For Western analysis, 10 µg protein samples were electrophoresed on NuPAGE 4–12% SDS-polyacrylamide gels (Invitrogen, Carlsbad, CA, USA) and transferred to PVDF membranes (Invitrogen). Primary antibodies were goat anti-TAOK3 (ab21205, ABCAM, Inc., Cambridge, MA) 1∶1000, rabbit anti-phospho-JNK1+JNK2+JNK3 (ab76572, ABCAM, INC.) 1∶1000, and mouse anti-GAPDH (Fisher Scientific, Pittsburgh, PA, USA), 1∶5000. Western blots were incubated with HRP-linked donkey anti-goat (Santa Cruz Biotechnology, Inc., Santa Cruz, CA, USA), HRP-linked donkey anti-rabbit or HRP-linked sheep anti-mouse secondary antibody (GE Healthcare, Biosciences, Piscataway, NJ, USA), 1∶10000, and processed with ECL Plus Western Blotting Detection System (GE Healthcare, Biosciences). Chemiluminescence was visualized and quantified using the Storm 660 phosphorimager system (Molecular Dynamics, Sunnyvale, CA, USA). For phospho-JNK experiments, wild-type and *Taok3Gt/+* mice were treated with vehicle (0.9% saline) or 4 g/kg ethanol (20% v/v in saline) via intraperitoneal injection, 5 minutes prior to decapitation and dissection of brain tissue for protein extraction. 3 animals per experimental group were utilized.

### Mouse Ethanol-induced Locomotor Stimulation

Mice for behavioral testing were bred in-house (*Taok3Gt*/+ and wild-type littermate controls) or obtained from the Jackson Laboratory (C57BL/6J mice for studies investigating the effects of SP600125 on ethanol clearance and consumption). Male mice were housed 3–5 per cage and were 10–14 weeks of age at the time of behavioral testing. Subjects were ethanol-naïve prior to each behavioral test.

Locomotor activity in response to ethanol was measured using 46×46 cm open field chambers (Med Associates, St. Albans, VT, USA). On the first day of testing, mice were allowed free access to the open field chambers for 1 hour, removed from the apparatus and administered a saline injection intraperitoneally, then returned to the chamber for an additional hour. On the second day of testing, the procedure was repeated with a 1 g/kg injection ethanol instead of saline. Horizontal distance traveled (in cm) was recorded in 5 minute bins for the duration of testing, with activity during the first 5 minutes post saline injection compared to activity during the first 5 minutes post ethanol injection.

### Mouse Loss-of-righting Reflex (LORR)

Ethanol (10% v/v in saline) was administered intraperitoneally at a dose of 4 g/kg. After injection, mice were placed on their backs and tested for LORR. The mouse was judged to have lost the righting reflex at the time when it could not right itself three times within 30 sec. When the animal was able to right itself three times within 30 sec it was deemed to have recovered. The duration of the LORR was calculated as the difference between when the reflex was lost and when it was recovered. For pharmacological studies, inhibitor JNK II (SP600125, Anthra[1,9-cd]pyrazol-6(2H)-one, 1,9-pyrazoloanthrone, VWR, Radnor, PA, USA) was dissolved in 5%Tween 80/0.9% NaCl and injected subcutaneously at a dose of 5 mg/kg, 30 min prior to ethanol treatment. This dose was selected empirically based on previous studies [38], [39], [40], [41].

### Mouse Ethanol Self-administration

Oral alcohol self-administration was examined using a limited access, drinking in the dark (DID) assay. One week prior to data collection, mice were singly housed and transferred to a reverse light-dark cycle. On day 8 of the experiment, water intake (ml) was measured for the 4-hour period beginning 4 hours after lights off and body weight (g) was recorded. Subsequently, mice were given access to a single bottle of 20% w/v ethanol during the same time of day, on alternate days for 12 days (6 days of ethanol access) and ethanol consumption was calculated. For pharmacological studies, subjects received a subcutaneous vehicle injection (Tween 80/0.9% NaCl) 10 min prior to ethanol availability on the first three days of ethanol access to acclimate subjects to injections. On subsequent ethanol access days subjects in the experimental group received a 5 mg/kg subcutaneous injection of inhibitor JNK II/Tween 80/0.9% NaCl, whereas control subjects continued to receive vehicle.

### Mouse Ethanol Metabolism and Clearance

Mice were injected with 4 g/kg of ethanol and tail blood samples (10 µl) were obtained at 10, 30, 60, 120 and 180 minutes to measure blood alcohol levels. For pharmacological studies, subjects received a 5 mg/kg subcutaneous injection of inhibitor JNK II/Tween 80/0.9% NaCl or vehicle 30 min prior to ethanol treatment. Blood ethanol content was assessed from serum using the Analox AM1 Analyzer (Analox Instruments, North Yorkshire, UK).

### Statistical Analysis

Data were analyzed using SigmaStat 3.1 (Systat Software, San Jose, CA, USA) or Prism (GraphPad, La Jolla CA). LORR data were analyzed by student’s *t* test. Ethanol consumption during DID experiments were analyzed by 2-way repeated-measures ANOVA for genotype X consumption (or JNK inhibitor treatment X consumption), followed by Newman-Keuls posthoc comparison. Similarly, ethanol clearance data were analyzed by 2-way repeated-measures ANOVA for genotype X time (or JNK inhibitor treatment X consumption), followed by Newman-Keuls posthoc comparison. mRNA and protein quantification in *Taok3Gt/+* mice was normalized to control littermates and GAPDH signal and compared by student’s *t* test. Data are presented as mean ± standard error of the mean.

## Results

### Mutation of JNK Pathway Genes Suppresses the Brain Structural Defects of *tao* Mutants

**Figure 1:**
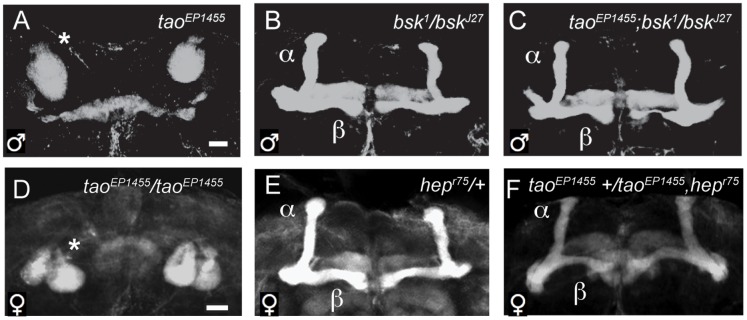
Mutations in *bsk* and *hep* suppress mushroom body morphology defects in *tao* mutants. *A–F* , Anterior projections of whole-mount adult brains stained with anti-FasII. ***A***, *tao^EP1455^* hemizygous male brain. Asterisk marks mass of axons in the posterior brain characteristic of *tao* mutants. ***B*** *, bsk^1^/bsk^J27^* male brain, with normal gross morphology of mushroom body α/β lobes. ***C***, Double mutant *tao^EP1455^; bsk^1^/bsk^J27^* male brain, with mushroom body α/β lobes indicated. ***D***, *tao^EP1455^/tao^EP1455^* homozygous female brain. ***E***, *hep^r75^/+*, and ***F***, *tao^EP1455^+/tao^EP1455^ hep^r75^* female brains. Scale bars = 15 µm.

Because both fly *tao* and mouse *Taok3* were identified as negative regulators of the JNK MAP kinase pathway signaling in cultured cells [23], [27], [30], [31], [42], we hypothesized that *tao* may act as a negative regulator of JNK signaling in the fly brain. Mutation of fly *tao* results in strong resistance to the locomotor stimulant properties of ethanol, and also results in highly penetrant structural defects in key neuropils of the central brain, including the mushroom bodies [22]. As a first test, we determined whether mutations in JNK pathway genes suppressed or enhanced the mushroom body structural defects of the loss of function allele *tao^EP1455^*, in which axons of mushroom body α/β neurons fail to form lobes [22], (Fig. 1*A, D*).

We examined the morphology of mushroom body α/β lobes in the brains of flies mutant for *tao* combined with loss-of-function alleles of the lone *Drosophila* JNK MAP kinase gene *basket* (*bsk)*. Heterozygous hypomorphic *bsk* alleles had little to no effect on the mushroom body morphology defects in male flies hemizygous for *tao^EP1455^* (data not shown). However, a viable combination of two hypomorphic alleles, *bsk^1^/bsk^J27^*, that did not affect mushroom body morphology (Fig. 1*B*), almost completely restored normal mushroom body morphology in *tao^EP1455^* mutants (Fig. 1*C*). We further examined the brain morphology of these *bsk* mutants by immunostaining with anti-tyrosine hydroxylase (TH), which marks dopaminergic neurons, specific sets of which control ethanol-induced hyperactivity [43], and observed no defects in cell number or anatomical projection (data not shown). To confirm that the JNK pathway was responsible, we tested mutations in the upstream JNK MAPK kinase *hemipterous* (*hep*). For this test we used females because hemizygous strong loss-of-function mutations of the X-linked *hep* are lethal to males. Like hemizygous males, *tao^EP1455^* homozygous females lack MB α/β lobes, displaying a “ball” of axons in the posterior of the brain near the MB calyx (Fig. 1*D*). We observed that heterozygotes for the null allele *hep^r75^* had normal α/β lobe morphology (Fig. 1*E*), and that heterozygous *hep^r75^* completely suppressed the mushroom body defects of homozygous *tao^EP1455^* females (Fig. 1*F*). These results indicate that loss-of-function mutants in JNK pathway components can suppress the *tao* mushroom body morphology defect, and suggest that *tao* acts as a suppressor of JNK pathway signaling in the nervous system.

### Mutations in the JNK MAPK Gene *basket* Affect Ethanol-induced Hyperactivity and Interact Genetically with *tao*

**Figure 2:**
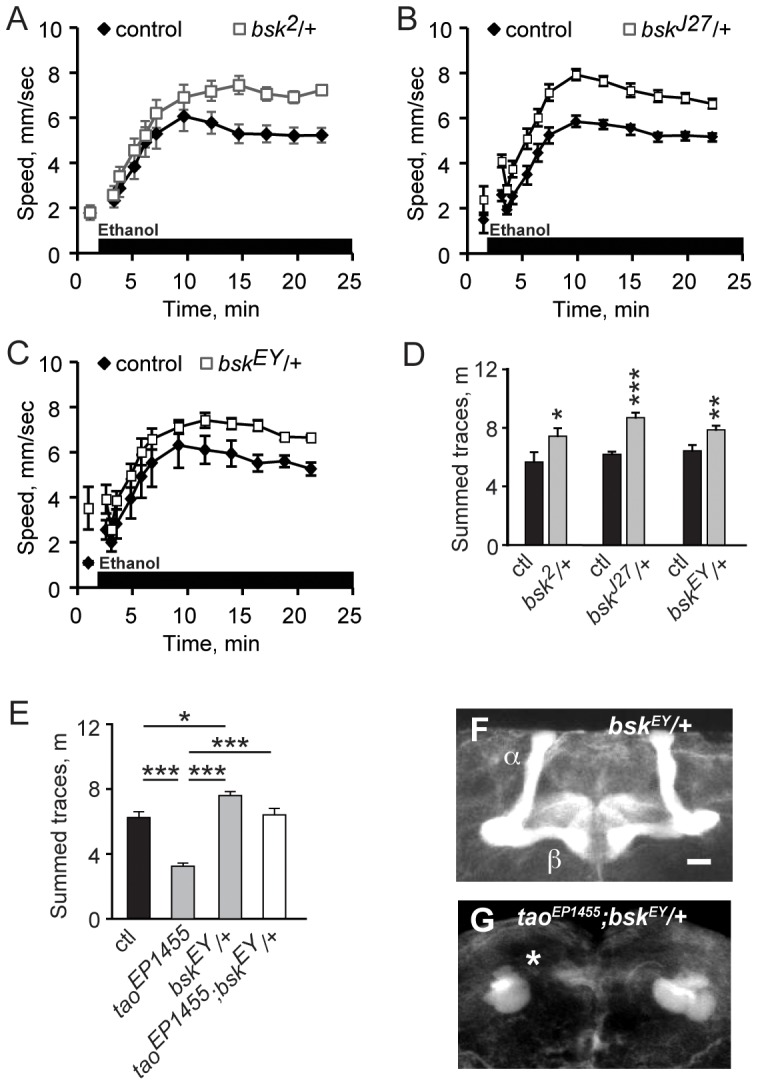
Genetic interaction between *tao* and *bsk*. ***A*** **,** Flies exposed to a continuous stream of ethanol vapor (black bar on x-axis) increase locomotor activity over time. ***A***, *bsk^2^*/+ ***B***, *bsk^J27^/+* and ***C***, *bsk^EY01915^/+* (“*bsk^EY”^* is *bsk^EY01915^*) mutants show enhanced ethanol-stimulated locomotion. Locomotor speed measured just prior to ethanol exposure is indicated at minute 1 for each graph. ***D***, Quantification of traces in ***A–C***, measured as the distance traveled during the hyperactive phase. ***E*** **,** Mutation of *bsk* suppressed the decreased ethanol-induced locomotor stimulation of *tao^EP1455^* flies. ***F***, *bsk^EY01915^* did not affect mushroom body morphology. Anterior projections of adult male wholemount brains stained with anti-FasII. α/β lobes are indicated. ***G***, Lack of suppression of mushroom body structural defects in *tao^EP1455^;bsk^EY01915^/+* double mutant. Asterisk indicates posterior axon mass characteristic of *tao^EP1455^* mutants. Ethanol absorption was unaltered in *bsk* mutants compared to the genetic background control strain (*p* = 0.5229, one-way ANOVA). Genotypes were compared using one-way ANOVA with Bonferroni correction. *p<0.05, **p<0.01, ***p<0.0001. Scale bars = 15 µm.

*tao* mutations result in strong resistance to the locomotor stimulant effects of acute ethanol exposure in flies [22]. To determine if decreased JNK signaling affects sensitivity to acute ethanol exposure, we tested male flies heterozygous for conventional loss-of function mutations in *bsk* [44], [45]. *bsk^1^/+* flies showed normal ethanol-induced hyperactivity, but *bsk^J27^*/+ and *bsk^2^*/+ heterozygotes as well as flies heterozygous for *bsk^EY01915^*, a P-element insertion in *bsk* that failed to complement the hypomorphic *bsk^1^* allele for lethality displayed increased ethanol-induced hyperactivity (Fig. 2*A–D*), suggesting that reduced JNK signaling increased sensitivity to the locomotor stimulant effects of ethanol. These mutants showed normal kinetics of ethanol accumulation, indicating that the *bsk* phenotype is not due to altered ethanol absorption or metabolism (Fig. 2 legend).

To test whether the negative genetic interaction between *tao* and JNK signaling extends to behavior, we assayed the effects of acute ethanol exposure on double mutant flies that were hemizygous for *tao^EP1455^* and heterozygous for *bsk^EY01915^.* The double mutant flies displayed increased ethanol-induced locomotion relative to flies singly mutant for *tao* (Fig. 2*E*), indicating that decreased JNK signaling was sufficient to restore normal hyperactivity to *tao*, and consistent with a negative regulatory interaction between *tao* and JNK signaling for ethanol behavior. Interestingly, heterozygous *bsk^EY01915^* did not suppress the MB morphology defect caused by *tao^EP1455^* (Fig. 2*F,G*): these flies completely lacked MB α/β lobes. These data indicate that the increase in hyperactivity observed in the *bsk* mutant is independent of any structural effect on the MB and suggest that JNK signaling may be required in one or more brain loci that compensate for the loss of the MB α/β lobes caused by the *tao^EP1455^* mutation.

### JNK Phosphatase Puckered Promotes Ethanol-induced Hyperactivity

Our finding that *bsk* mutants exhibit increased sensitivity to the locomotor stimulant effects of acute ethanol exposure suggested that the JNK signaling pathway is important for this behavior. Expression of the MAPK phosphatase Puckered (Puc) is induced by Bsk activation, and subsequently Puc dephosphorylates and inactivates Bsk in a negative feedback loop [46], [47]. Consequently, loss of *puc* function increases JNK activation in response to upstream signals. Microarray studies indicate that *puc* expression is induced in response to acute ethanol exposure, with kinetics suggesting that the activation of the JNK pathway might play a role in ethanol-induced behavior [35].

**Figure 3:**
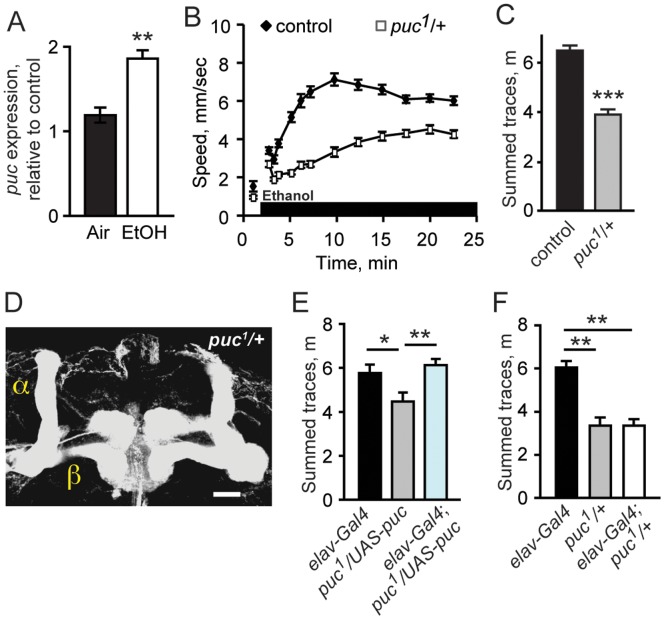
*puc* is required in neurons for ethanol-induced locomotor stimulation. ***A***, Quantitative PCR detection of *puc* expression levels in fly head extracts following 30 min ethanol or air control exposure. **p = 0.0024 *t* test. ***B***, Reduced ethanol-induced locomotor stimulation in *puc^1^/+* flies. ***C***, Quantification of traces shown in ***B***. ***p<0.0001 *t* test. Ethanol absorption of *puc1/+* was unaffected (*p* = 0.9607 *t* test). ***D*** **,** Mushroom body morphology of *puc^1^/+* mutants was normal. Scale bar = 10 µm. ***E,*** Decreased ethanol-induced locomotor stimulation of *puc^1^/+* mutants rescued by neuron-specific expression of *puc*. *puc^1^/+* mutants carrying a *UAS-puc* transgene were crossed to the *elav^C155^-Gal4* to restore *puc* expression levels specifically in neurons. ***F***, *elav^C155^-Gal4* did not increase ethanol-induced locomotor stimulation in *puc^1^/+* mutants.

To confirm ethanol-induction of *puc* transcription, we exposed wild-type male flies to ethanol vapor or air and measured *puc* RNA levels in whole head extracts using quantitative PCR analysis. We found that *puc* levels were increased approximately two-fold in ethanol-treated flies, consistent with activation of JNK signaling by acute ethanol exposure (Fig. 3*A*). We next tested whether decreased *puc* expression affected ethanol-induced hyperactivity. Flies heterozygous for *puc^1^*, a strong loss-of-function allele of *puc*, showed greatly reduced ethanol-induced hyperactivity (Fig. 3*B,C*), indicating that reduced *puc* activity and increased activation of the JNK pathway reduced this behavior, consistent with the increased hyperactivity observed in *bsk* mutants. As with *bsk* mutants, we examined brain morphology in *puc^1^/+* flies using anti-FasII, and anti-TH staining, and observed no defects (Fig. 3*D*, data not shown). Further, *puc^1^/+* flies had normal kinetics of ethanol accumulation (Fig. 3 legend). We conclude that increased JNK signaling decreases sensitivity to the locomotor stimulant properties of ethanol in *Drosophila*. While a developmental role for JNK signaling in ethanol-induced behavior cannot be ruled out, the induction of JNK pathway activity by ethanol exposure suggests that the pathway more directly modulates ethanol-induced behaviors.

### Neuronal Expression of *puc* Rescues Ethanol-induced Hyperactivity in *puc* Mutants

Tao activity is both necessary and sufficient in neurons in flies for increasing sensitivity to the locomotor stimulant properties of ethanol [22], and our results indicate that JNK signaling acts downstream of Tao. To investigate whether JNK acts in neurons to regulate ethanol responses, we used the Gal4-UAS heterologous expression system to test if neuron-specific expression of *puc* could rescue the ethanol phenotype of *puc^1^/+* flies. We crossed flies carrying *puc^1^* and a *UAS-puc* transgene to the pan-neuronal driver line *elav^C155^-Gal4,* and compared the ethanol-induced hyperactivity of the progeny to single-transgenic controls. We found that ethanol-induced hyperactivity was restored in *puc^1^/+* mutants with *elav^C155^-Gal4* and *UAS-puc*, showing that the behavioral defect of *puc^1^/+* flies can be rescued with neuron-specific expression of *puc* (Fig. 3*E*). *elav^C155^-Gal4* did not affect ethanol-induced hyperactivity of *puc^1^/+* flies in the absence of the *UAS-puc* transgene, indicating that expression of Gal4 alone in neurons was not responsible for the rescue of the *puc^1^/+* phenotype (Fig. 3*F*). Thus expression of *puc* solely in neurons is sufficient to promote ethanol-induced hyperactivity.

**Figure 4:**
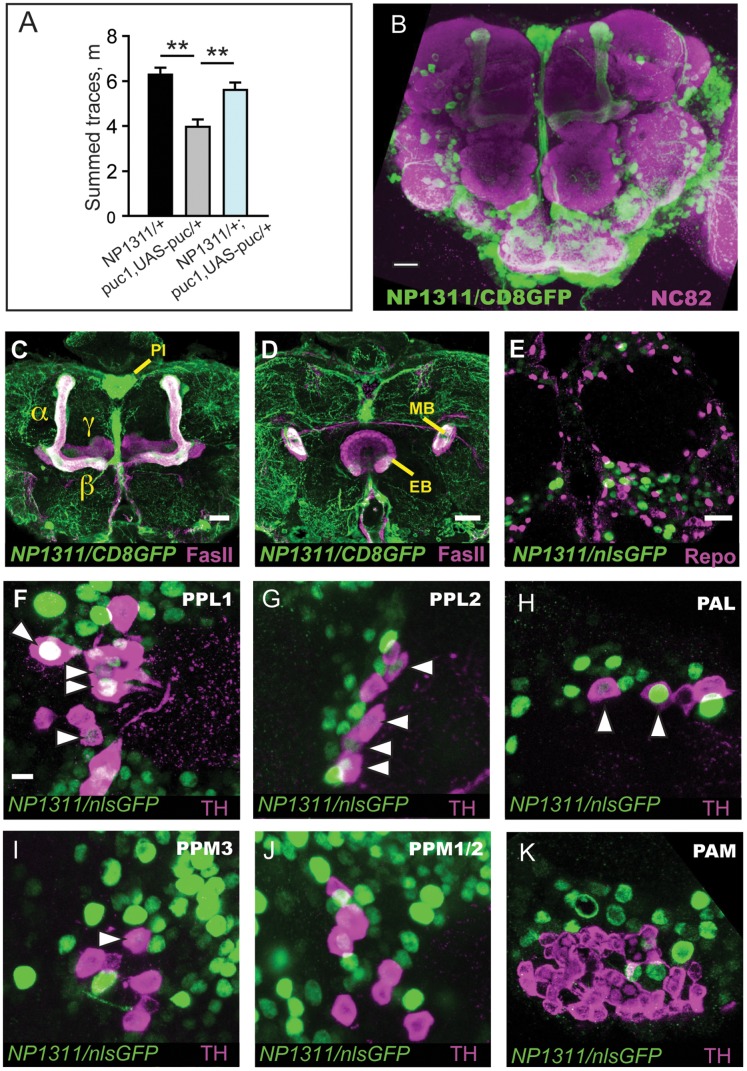
Expression pattern of a *puc* reporter line in the central brain. ***A***, Rescue of decreased *puc^1^/+* ethanol-induced locomotor stimulation in the *NP1311* expression pattern. Rescue genotypes were compared using one-way ANOVA with Bonferroni correction. *p<0.05, **p<0.01. ***B–K***, Expression pattern of *NP1311 puc* enhancer trap detected with *UAS-GFP*. ***B*** *,* Confocal projection of the central brain, with NP1311 expression visualized with membrane-bound GFP (UAS-CD8.GFP, counterstained with NC82 to visualized neuropil. ***C***, Projection showing mushroom body α/β and γ lobes, counterstained with anti-FasII. PI: pars intercerebralis. ***D***, Projection showing lack of expression in the ellipsoid body (EB), counterstained with anti-FasII. ***E***, *NP1311* is neuron-specific. Individual cells in *NP1311*/+; *UAS-nls.GFP*/+ brains counterstained with anti-Repo to mark glia. ***F–K***, NP1311 expression in dopaminergic neuron clusters. Arrowheads indicate cells with overlapping nls.GFP (green) and tyrosine hydroxylase (TH, magenta) staining. ***F*** *,* PPL1 cluster, ***G*** *,* PPL2, ***H*** *,* PAL, and ***I*** *,* PPM3. No overlap was observed in ***J*** *,* PPM1/2, or ***K*** *,* PAM. Scale bars = 20 µm (***B***), 10 µm (***C,D***), or 5 µm (***E*** **,** ***F-K***).

To define the site of action for *puc* for ethanol-induced hyperactivity, we examined the expression pattern of *NP1311*, a Gal4 enhancer trap transposon inserted in the third intron of the *puc* locus. We asked whether expression of *puc* specifically in this limited pattern was sufficient to rescue the behavioral phenotype of *puc^1^/+* mutants. Flies heterozygous for *NP1311* displayed normal levels of ethanol-induced hyperactivity, and restricted expression of *puc* in the *NP1311* pattern was sufficient to restore ethanol-stimulated locomotion to control levels in *puc^1^*/+ mutants (Fig. *4A*). These results indicate that *puc* expression in the pattern of *NP1311* expression is sufficient to promote the locomotor stimulant properties of ethanol.

To define the expression pattern of *NP1311*, brains of *NP1311*/+;*UAS-CD8.GFP*/+ and *NP1311*/+;*UAS-nls.GFP*/+ flies were dissected and GFP expression visualized *NP1311* drove GFP expression strongly in a number of adult brain loci, including the α/β neurons of the mushroom bodies (Fig. 4*B,C*). However, GFP expression was absent from neurons of the ellipsoid body, a brain region that is required to promote ethanol-stimulated locomotion [43], (Fig. 4*D*). Expression was seen in a number of dopaminergic neurons, including cells in the PPL1 (Fig. 4*F*), PPL2 (Fig. 4*G*), and PAL (Fig. 4*H*) clusters. Some expression was seen also in the PPM3 cluster (Fig. 4*I*), which also affects ethanol-induced locomotion. No expression was seen in the PPM1/2 (Fig. 4*J*) or PAM (Fig. 4*K*) dopaminergic clusters. No expression was observed in glia (Fig. *4E*). The *NP1311* enhancer trap likely captures a significant portion of the endogenous expression pattern of *puc* [48].

### Disruption of *Taok3* in Mice Alters Acute Sensitivity to Ethanol

**Figure 5:**
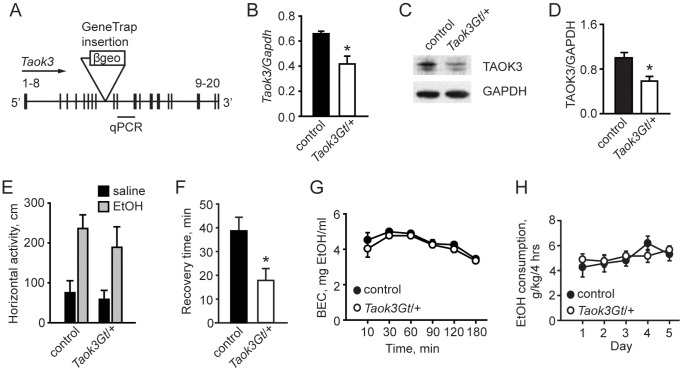
Characterization of *Taok3Gt/+* mice. ***A***, Schematic of the *Taok3* locus with the genetrap insertion located between exons 8 and 9 of the gene. Exon numbers are indicated. “β geo” = β galactosidase/neomycin resistance markers within the genetrap insertion. ***B***, Quantitative PCR data showing reduced *Taok3* transcript in brain tissue of *Taok3Gt/+* mice compared to controls. *p<0.05 *t* test. ***C, D***, Western blot analysis of brain lysates from *Taok3Gt/+* and wild-type control mice showing reduced TAOK3 protein levels in mutants. *p<0.05 *t* test. ***E***, *Taok3Gt/+* mice exhibit normal basal activity and locomotor stimulation in response to 1 g/kg ethanol. *p* = 0.27 ANOVA. ***F***, *Taok3Gt/+* mice are resistant to ethanol-induced sedation in the LORR assay following treatment with 4 g/kg ethanol. *p<0.05 *t* test. ***G***, Ethanol absorption/clearance is unaffected by the *Taok3Gt* allele, BEC = blood ethanol content. *p* = 0.07, repeated measures ANOVA. ***H***, Voluntary ethanol consumption is normal in *Taok3Gt/+* mice. *p* = 0.85, repeated measures ANOVA.

Because *tao* interacts genetically with the JNK pathway and because JNK pathway activation affects ethanol-induced behavior in *Drosophila* we investigated whether these relationships were conserved in ethanol-dependent behaviors in mice. The Tao kinase family is represented in vertebrates by three orthologous genes, *Taok1, Taok2* and *Taok3* [24], [32]. Based on our observation that *Drosophila tao* interacts negatively with the JNK MAPK genes *bsk* and *hep*, we chose to investigate *Taok3*, the only TAOK that can inhibit JNK activity in cultured cells [30]. We generated *Taok3* genetrap mice using the cell line RRK451 from the International Gene Trap Consortium (http://www.genetrap.org/). This cell line contains a genetrap insertion in the eighth intron of the *Taok3* gene and is predicted to result in a fusion transcript of exons 1–8 of *Taok3* with the gene encoding β-galactosidase, truncating the endogenous *Taok3* transcript within sequence encoding a portion of the kinase domain (Fig. 5*A*). Mice heterozygous for the *Taok3* genetrap allele (*Taok3Gt/+*) were viable and normal in appearance. However, we failed to recover homozygous *Taok3Gt/Gt* mice, suggesting that disruption of both copies of *Taok3* results in embryonic lethality and that the *Taok3Gt* allele may be hypomorphic or null.

To further characterize *Taok3* gene disruption by the genetrap allele, we measured *Taok3* transcript levels in brain tissue obtained from adult *Taok3Gt/+* mice by quantitative PCR and observed a 40% reduction of endogenous *Taok3* transcript (Fig. 5*B*). Similarly, we observed a 50% reduction in TAOK3 protein levels in brain tissue from *Taok3Gt/+* mice by Western analysis (Fig. 5*C,D*), indicating that the genetrap insertion effectively disrupted the *Taok3* gene. We also observed wild-type levels of TAOK1 and TAOK2 in brain tissue from *Taok3Gt/+* mice (data not shown), indicating that disruption of one copy of the *Taok3* gene did not result in compensatory changes in protein levels of TAOK1 or TAOK2.

Because disruption of the *Drosophila tao* gene strongly reduced the locomotor stimulant properties of acute ethanol exposure [22], we assessed the behavioral response of *Taok3Gt/+* mice to ethanol in a battery of tests. We first assessed *Taok3Gt/+* and wild-type control mice for hyperactivity induced by a 1 g/kg dose of ethanol. We observed a normal response (Fig. 5*E*), indicating that disruption of one copy of the *Taok3* gene was not sufficient to affect ethanol-stimulated locomotion.

In contrast, when we tested *Taok3Gt/+* mice in the loss-of-righting reflex (LORR) assay, a test of the sedative-hypnotic effects of ethanol, we observed a significant decrease in recovery time (Fig. 5*F*). We assessed ethanol metabolism/clearance of the same 4 g/kg dose ethanol in a drug-naïve group of animals and failed to detect a difference between genotypes (Fig. 5*G*), suggesting that the resistance of *Taok3Gt/+* mice to the sedative properties of ethanol was not the result of altered ethanol pharmacokinetics.

Studies in humans and in rodent models suggested that initial sensitivity to ethanol may predict subsequent ethanol consumption [2], [49], [50], [51], [52], [53], [54], [55], [56]. However, using a limited access paradigm in which mice gained access to a single 20% w/v solution of ethanol for 4 hours during the dark or active phase of their circadian cycle, we did not detect a genotypic difference in ethanol consumption (Fig. 5*H*). Thus, reduction of *Taok3* expression conferred resistance to the sedative effects of ethanol, but it did not alter ethanol consumption or ethanol-stimulated locomotion.

### JNK is Negatively Regulated by TAOK3 in Mice and is Activated in the Brain by Ethanol

**Figure 6:**
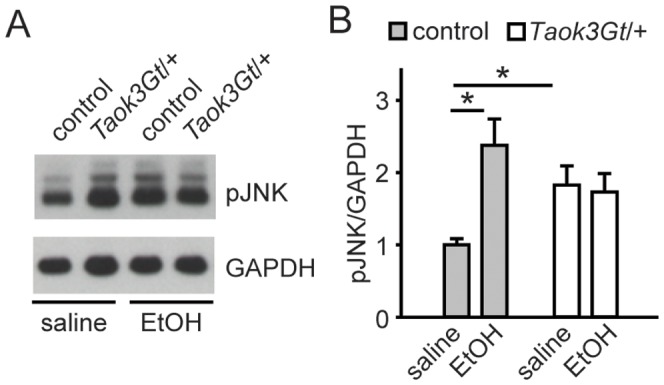
JNK activity is induced by ethanol ***A, B.*** Western blot analysis of brain lysates from *Taok3Gt/+* and wild-type control mice showing induction of phospho-JNK by ethanol in wild-type mice and increased phospho-JNK levels in brains of saline-treated *Taok3Gt/+* mice relative to controls. *p<0.05 *t* test. Ethanol induction of phospho-JNK is occluded in *Taok3Gt/+* mice.

Mammalian cell culture experiments demonstrated that TAOK3 is a negative regulator of JNK activity; indeed, TAOK3 initially was named “JIK” for “JNK Inhibitor Kinase” [30]. To determine if TAOK3 inhibits JNK in* vivo*, we performed Western analysis using total brain extracts from *Taok3Gt/+* and control mice. We found that phospho-JNK levels were constitutively increased in brains of *Taok3Gt/+* mice, suggesting that TAOK3 negatively regulates JNK *in vivo* (Fig. 6*A,B*).

JNK activity in the brain might also be regulated by ethanol exposure [21], [57], [58], [59], [60], [61], [62], [63], [64], [65]. We examined whether JNK activation was affected by treating mice with an acute, sedating dose of ethanol (4 g/kg). We observed an increase in phospho-JNK upon ethanol treatment in the brains of wild-type mice compared to saline-treated controls, indicating that acute ethanol treatment increased JNK activity in the brain (Fig. 6*A,B*). However, acute ethanol treatment did not increase the level of phospho-JNK in brains of *Taok3Gt/+* mice relative to controls (Fig. 6*A,B*). Thus, while reduced TAOK3 levels increased constitutive JNK pathway activity in the brain, it prevented increased JNK activation that was elicited by acute ethanol exposure.

### JNK Inhibition Confers Resistance to Ethanol-induced Sedation in Mice

**Figure 7:**
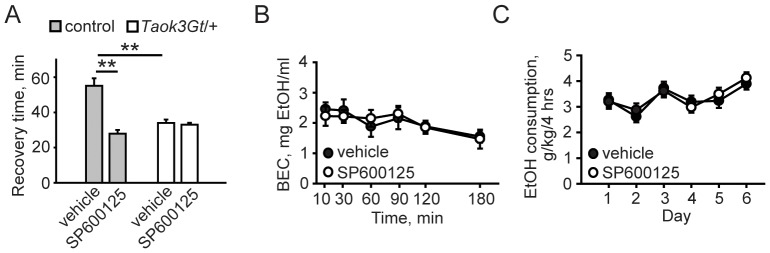
JNK activity modulates behavioral sensitivity to ethanol in mice. ***A***, Pharmacological inhibition of JNK confers resistance to ethanol-induced sedation in wild-type but not *Taok3Gt/+* mice. Mice were treated subcutaneously with vehicle or 5 mg/kg JNK inhibitor SP600125, 30 minutes prior to the LORR assay. **p<0.01, *t* test. ***B***, SP600125 (5 mg/kg) does not alter ethanol pharmacokinetics. *p* = 0.94 repeated measures ANOVA. BEC = blood ethanol content. ***C***, SP600125 (5 mg/kg) does not affect voluntary ethanol consumption. *p* = 0.73 repeated measures ANOVA. All mice received vehicle injections on days 1–3 before assignment to control or experimental groups.

Based on our data suggesting that TAOK3 promotes ethanol-induced sedation and that ethanol increases activated JNK in a TAOK3-dependent manner, we predicted that acute inhibition of the JNK pathway might affect ethanol-induced sedation. We tested this hypothesis by administering to wild-type mice 5 mg/kg of SP600125, which acts as a reversible ATP-competitive inhibitor of JNK MAP kinases [66], 30 minutes prior to the LORR assay. We found that SP600125 treatment conferred resistance to the sedating effects of ethanol in wild-type mice (Fig. 7*A*). In contrast, we found that SP600125 treatment failed to alter the duration of ethanol-induced sedation in *Taok3Gt/+* mice (Fig. 7*A*). The same dose of JNK inhibitor did not alter clearance of 4 g/kg ethanol (Fig. 7*B*), suggesting that the effect of SP600125 on ethanol-induced sedation was not the result of altered ethanol pharmacokinetics. We subsequently tested whether SP600125 affected voluntary ethanol consumption in the limited access assay, but observed no significant effect of treatment (Fig. 7*C*).

## Discussion

We previously demonstrated that the Ste20 Kinase TAO was required in the fly brain for both establishing mushroom body structure and promoting sensitivity to the locomotor stimulant properties of acute ethanol exposure. We now show that 1) the TAO mouse homolog TAOK3 promotes sensitivity to acute ethanol sedation in mice, 2) both fly TAO and mouse TAOK3 inhibit JNK signaling in vivo, 3) JNK signaling affects behavioral responses to acute ethanol exposure in both flies and mice, 4) the role of JNK in ethanol behavior localizes to specific neurons in the fly brain, and 5) the mechanism by which the fly JNK gene *bsk* interacts with *tao* for ethanol behavior is independent of the role of *tao* in mushroom body structure. We propose that TAO/TAOK3 regulates JNK activity to promote sensitivity to the acute behavioral effects of ethanol. The role of JNK signaling in cell death induced by ethanol has been well documented: here we observe behavioral phenotypes in mutant flies at 2–4 fold lower ethanol concentrations and shorter exposures that do not result in cell death in *Drosophila* [67]. Recent cell culture and genetic interaction experiments allow us to propose a signaling framework for ethanol behavioral responses that includes TAO/TAOK3, JNK, and the kinase Par-1.

While manipulation of JNK pathway signaling caused ethanol-induced behavioral phenotypes in both flies and mice, the behaviors affected were different in these organisms. In *Drosophila*, JNK signaling may affect ethanol-induced locomotion specifically. A previous study did not detect changes in acute ethanol sedation sensitivity in flies that overexpressed wild-type, activated, and dominant negative JNK pathway proteins in the nervous system [4]. We similarly observed no effect on sedation sensitivity of the JNK pathway hypomorphic mutations (data not shown). Greater reductions in *puc* and *bsk* expression did result in altered ethanol sedation sensitivity, however these mutants also exhibited deficits in general locomotion, complicating the interpretation of ethanol behavioral phenotypes. RNAi directed against *bsk* has been reported to reduce sleep by increasing locomotion [68]. We believe, however, that the increase in ethanol-induced locomotion is mechanistically distinct, because in two of the three *bsk* alleles we tested we did not observe increased pre-exposure locomotor activity.

Conversely, mouse *Taok3* mutants with constitutively activated JNK showed altered ethanol-induced sedation, but showed no effect on ethanol-induced locomotor activation. Acute disruption of JNK signaling in mice by the inhibitor SP600125 also affected ethanol-induced sedation, though we have not yet tested its effects on ethanol-induced locomotor activation. Gene family diversification may explain the species specific phenotypes: in mammals TAOK1 or TAOK2 may functionally compensate for TAOK3 with respect to ethanol-stimulated locomotion. As an initial test of this hypothesis, we observed unaltered ethanol-stimulated locomotion in mice in which both copies of the *Taok2* gene were disrupted [69]. The analysis of compound *Taok1*/*Taok2*/*Taok3* mutants may help clarify to what extent, if any, these genes are functionally redundant with respect to ethanol-stimulated locomotion. The JNK and other MAPK signaling pathway gene families also increase in complexity from flies to mice, such that additional positive and negative signaling activities may mask behaviorally relevant actions of specific JNK isoforms. Alternatively, the TAO/JNK signaling pathway may be used differently in different organisms.

We identified the MAPK phosphatase Puc as a key mediator of JNK pathway effects on ethanol-induced behavior in *Drosophila*. *puc* expression was induced upon ethanol exposure, decreased *puc* expression conferred resistance to the locomotor stimulating effects of ethanol, and the behavioral actions of *puc* localized to the nervous system. Importantly, *puc* expression induction indicates active JNK signaling [47], and is consistent with the increased phospho-JNK observed in the brains of ethanol treated mice. Combined with the behavioral phenotypes of *bsk*/JNK and *hep*/JNKK, the *puc* findings indicate that JNK activity normally promotes ethanol-stimulated locomotion in *Drosophila*. Puc is related to the DUSP family of mammalian MAPK phosphatases that are capable of dephosphorylating MAP kinases of all three families [70], [71], [72]. The modulation of JNK activity in mammals may be accomplished by multiple regulatory phosphatases in a more complex manner than in flies.

We also found that expression of Puc in a limited set of neurons is sufficient to promote sensitivity to the locomotor stimulant properties of ethanol. The expression pattern of the *puc NP1311* Gal4 enhancer trap used for genetic rescue includes the MB α/β neurons, and the function of these neurons is required for promoting ethanol-stimulated locomotion [22]. However, *bsk* mutations can restore ethanol-induced hyperactivity in *tao* mutants lacking the MB α/β axon lobes, implying that the MB are not the primary locus at which JNK signaling affects ethanol-stimulated locomotion. Instead, JNK signaling may act in a separate neural locus that can compensate for function of the MB in this behavior. The R2/4 neurons of the ellipsoid body are also required for normal ethanol-stimulated locomotion [43], however these neurons are not included in the *NP1311* expression pattern. The *NP1311* pattern does encompass some dopaminergic neurons, including a small number of neurons in the PPM3 cluster, which are known to mediate ethanol-stimulated hyperactivity [43]. *NP1311* also expresses strongly in neurons of the *pars intercerebralis*, which affect ethanol-induced sedation sensitivity [73]. However, the circuitry governing ethanol-induced behaviors is poorly defined, and more detailed mapping of the requirement for JNK signaling using newer Gal4 enhancer traps that more sparsely label the nervous system [74] will be necessary to resolve the loci of JNK activity that affect behavior in *Drosophila*.

In mice both pharmacological inhibition of the JNK pathway and a 50% reduction in *Taok3* expression that increases activated JNK levels speeded recovery from ethanol-induced sedation. While these experiments clearly implicate JNK signaling in ethanol sensitivity in mice, a simple relationship between the level of activated JNK in the mouse brain and ethanol-induced sedation may not exist. However, *Taok3* mutant mice also failed to increase levels of activated JNK upon exposure to ethanol. It may be, therefore, that ethanol-induced behavior is affected by the net increase in activated JNK level over baseline, rather than by its absolute level. Consistent with this idea is the observation that *Taok3Gt/+* mice are resistant to the effects of SP600125 on ethanol-induced sedation and provides additional evidence that TAOK3 signals through JNK rather than parallel pathways to regulate ethanol-induced sedation. Alternatively, it is possible that *Taok3* mutations specifically affect the activation state of particular JNK isoforms, while acute inhibition of all JNK isoforms by SP600125 has a different aggregate effect on behavior.

Repeated high level (binge) or chronic ethanol intake induces oxidative stress that is linked to tissue damage in organs including the liver, and it is well established that oxidative stress induces JNK signaling [75]. Similar ethanol exposure conditions can also lead to neuronal death through less well defined mechanisms that may include oxidative stress, excitotoxicity, or thiamine deficiency [76], [77]. The ethanol concentrations that resulted in *tao*/*Taok3* and JNK pathway activation and behavioral phenotypes in mutants to a single acute exposure were too low to result in tissue damage [67], thus it is likely that a distinct JNK signaling mechanism is engaged. It remains possible, however, that acute ethanol exposure results in a neuronal oxidative stress response that is below the threshold for tissue damage, but that is sufficient to affect JNK signaling and behavior. Oxidative stress has been proposed to contribute to both normal synaptic development and synaptic plasticity [78]. Further, evidence in flies suggests that JNK signaling can affect both neuronal development and synaptic morphology [79], [80], [81].

Decreased *Drosophila par-1* expression also suppresses the structural and ethanol behavioral phenotypes of *tao* mutants [22]. This raises the possibility that Par-1 and the JNK pathway may define a signaling pathway with TAO. Both Par-1 and JNK are implicated in the planar polarity (PP) pathway that controls oriented growth of cells in tissue morphogenesis [82], [83], [84]. The core constituents of the PP pathway also control multiple steps of neuronal development, including neurite outgrowth, axon pathfinding, and axon branching [85], [86], [87]. Accordingly, the PP pathway is required for the appropriate development of the *Drosophila* mushroom bodies [87]. Furthermore, behavioral roles were recently found for specific genes in the core PP pathway, including susceptibility to seizures and to social defeat stress [88], [89], and core PP pathway genes are expressed in the adult nervous system. Members of the TAO kinase family interact with both PAR-1 and the JNK pathway [23], [27], [30], [31], [90], however a direct connection of TAO kinases to the PP pathway has not been established. At a cellular level Par-1, TAO kinases, and the JNK pathway all regulate the structural dynamics of the cytoskeleton [91], [92], and may exert these effects through the microtubule binding protein Tau [22], [93], [94], [95]. Indeed, the cytoskeleton is markedly rearranged following acute ethanol exposure [96], [97]. Although we failed to detect gross morphological defects in brain structure of *Taok3Gt/+* mice by immunohistological analysis (data not shown), we can not exclude the possibility that anomalies in brain morphology and/or neuronal connectivity underlie the ethanol sensitivity phenotype of these mice.
